# 776 Use of Paraffin Wax During Occupational Therapy Treatment of Burn Survivors

**DOI:** 10.1093/jbcr/irae036.317

**Published:** 2024-04-17

**Authors:** Kristin Rainey

**Affiliations:** Medical City Plano, Bells, TX

## Abstract

**Introduction:**

Burn survivors face long and often painful rehabilitation after injury. Unfortunately, the severe pain from burn injuries can lead to chronic pain and stress-related disorders. Occupational therapists work with burn survivors to improve movement and reduce negative impacts of scars to increase independence with self-care tasks, return to work and hobbies, and facilitate social participation. Paraffin wax is used as a modality by occupational therapists to decrease pain and assist with soft issue mobilization. It is imperative to determine if paraffin is beneficial specifically for burn injuries to facilitate physiological and psychological recovery.

**Methods:**

Low melting point paraffin wax at a temperature between 120-130 degrees without scent was chosen to prevent any burns or allergic reactions. Inclusion criteria was for subjects over the age of 18 years and all wounds on extremity being assessed must have been closed. Active range of motion (AROM) and passive range of motion (PROM) were assessed as well as pain score. The patient then washed the extremity with soap and water, dried, placed into the paraffin bath, dipped four times, then placed into a plastic liner and wrapped in a towel for 10 minutes. After the treatment was complete, ROM and pain were assessed and the patient was offered an opportunity to provide any feedback or comments.

**Results:**

A total of 7 burn survivors were identified and chosen for participation in the study. All patients showed improvement of AROM and PROM measurements of the tested extremity. Pain was reduced with every patient by an average of 30%. Every patient reported less pain and better movement, subjectively. Most patients reported less fear or anxiety. 42% of patients felt like their scar was softer after the paraffin treatment. Unfortunately, the study has a limited trial size due to requirement of completely closed wounds with continued follow up by the patient in clinic. Many patients with closed wounds do not follow up in the clinic on a regular basis. Interestingly, women reported more psychological benefits and also more likely to report a plan to continue using paraffin at home, compared to the men. AROM and PROM improved consistently within each session after paraffin was used, however week to week improvement was more variable between patients. This could be due to varying compliance of each individual’s home exercise program.

**Conclusions:**

Paraffin wax can be beneficial during occupational therapy for soft tissue mobilization of burn scars to increase range of motion and reduce pain and anxiety. Utilizing paraffin wax as a modality helps to reduce physiological and psychological barriers of traditional occupational therapy interventions and should be considered as an adjunct treatment.

**Applicability of Research to Practice:**

While the use of paraffin has been studied for treating conditions such as arthritis, scleroderma, and stroke, there is limited research on the use of paraffin for treating burn scars.

